# Women's non-farm employment and household dietary quality in rural China: evidence from a national rural health perspective

**DOI:** 10.3389/fpubh.2026.1910911

**Published:** 2026-08-31

**Authors:** Yuan Gao, Gangyi Wang, Xiaochen Zhang

**Affiliations:** School of Economics and Management, Northeast Agricultural University, Harbin, China

**Keywords:** dietary quality, household resource allocation, nutritional welfare, rural population, social determinants of health, women's employment

## Abstract

**Background:**

Improving dietary quality and reducing nutrition-related inequalities remain important public health challenges in rural populations. Women often play central roles in household food purchasing, meal preparation, and resource allocation, suggesting that women's employment transitions may be closely linked to household nutritional outcomes. This study examines the association between married women's non-farm employment and household dietary quality in rural China and explores potential pathways underlying this relationship.

**Methods:**

Data were drawn from 1,723 rural households in the China Rural Revitalization Survey (CRRS). Household dietary quality was assessed using the China Dietary Balance Index-22 (DBI-22), including the High Bound Score (HBS), Low Bound Score (LBS), and Diet Quality Distance (DQD), which capture excessive intake, insufficient intake, and overall dietary imbalance, respectively. Regression models, instrumental variable estimation, conditional mixed process estimation, propensity score matching, sensitivity analyses, subgroup analyses, and pathway analyses were used to examine the relationship between married women's non-farm employment and household dietary quality.

**Results:**

Married women's non-farm employment was significantly associated with better household dietary quality. Specifically, women's employment was associated with lower LBS and DQD, indicating reduced insufficient dietary intake and overall dietary imbalance, while no significant association was observed with HBS. The findings remained stable across sensitivity analyses, instrumental variable estimation, and propensity score matching. Pathway analyses suggest that the association may operate through increased household income, improved intra-household resource allocation, and changes in food consumption structure. Subgroup analyses further showed that the association was more pronounced among households with stronger nutritional demands, lower income levels, and greater market constraints.

**Conclusion:**

Married women's non-farm employment is an important social determinant of household nutritional welfare in rural China. Promoting gender-inclusive employment opportunities may contribute to improving dietary quality and reducing nutrition-related inequalities among rural populations.

## 1 Introduction

Dietary quality is a fundamental determinant of population health and an important dimension of household nutritional welfare. Poor dietary quality has been closely associated with chronic diseases, malnutrition, and long-term health inequalities, particularly among vulnerable populations experiencing rapid socioeconomic transformation (1, 2). From a public health perspective, dietary behaviors are not merely individual choices but are shaped by broader socioeconomic conditions, food systems, and household decision-making processes (3, 4). Meanwhile, the global nutrition transition has generated increasingly complex dietary challenges, with many societies simultaneously facing insufficient nutrient intake, dietary imbalance, and emerging risks of excessive consumption (5–7). Importantly, household dietary outcomes are closely related to intra-household resource allocation and gender roles. In rural households, women often play central roles in food purchasing, meal preparation, and nutrition-related management, suggesting that changes in women's socioeconomic participation may have important implications for household dietary quality.

In rural China, dietary transformation has been closely associated with changes in household livelihood strategies and food environments. In China, rapid economic development and food system transformation have shifted rural diets from traditional staple-oriented patterns toward more diversified consumption structures; however, disparities in dietary quality remain evident across households and regions (8–10). Existing studies have demonstrated that income growth can improve households' ability to access higher-quality foods, while food market conditions and local food environments also play important roles in shaping dietary choices and nutritional outcomes (11–13). However, household dietary quality is not determined solely by income levels. Household demographic characteristics, dietary knowledge, consumption preferences, and intra-household allocation mechanisms also influence food consumption decisions and nutritional welfare (14–17). Therefore, understanding the socioeconomic and household mechanisms underlying dietary quality improvement is essential, particularly in the context of ongoing rural livelihood transformation.

Among the various socioeconomic factors shaping household dietary outcomes, women's employment transition represents an important but insufficiently explored dimension. In rural China, women's labor participation has been substantially reshaped by rural industrialization, labor migration, non-farm employment opportunities, and changes in household livelihood strategies (18–21). Women's participation in non-farm employment may improve household welfare by increasing cash income, diversifying income sources, and enhancing households' ability to access higher-quality food products (22–25). Meanwhile, women's employment may also reshape intra-household labor allocation and decision-making processes because women's economic contribution can influence their bargaining position and participation in household resource allocation. Recent studies further suggest that women's employment transitions may affect household production arrangements, energy use, and health-related behaviors, indicating that female labor participation has broader implications beyond economic outcomes (26). However, women's employment may also generate potential trade-offs, as increased participation in market work may reduce time available for food preparation, caregiving, and household nutrition management. Therefore, the relationship between women's non-farm employment and household dietary quality remains theoretically ambiguous and requires further empirical investigation.

In this study, we focus specifically on married women rather than women in general because household dietary decisions and nutritional welfare are closely embedded within family structures. In rural households, married women often undertake major responsibilities related to food purchasing, meal preparation, and family nutrition management. At the same time, married women are typically more directly involved in intra-household resource allocation and welfare decisions compared with unmarried women, making their employment transitions more closely connected with household consumption behaviors and nutritional outcomes. Moreover, focusing on married women helps reduce heterogeneity arising from differences in household roles, living arrangements, and life-cycle stages, allowing a clearer examination of how women's economic participation influences household dietary quality.

The relationship between women's employment and household dietary quality is further complicated by gendered resource allocation. Previous studies have shown that women's empowerment and participation in household decision-making can improve food security, dietary diversity, and nutritional outcomes by increasing the allocation of resources toward health-related expenditures (27–30). However, these positive effects are not automatic, as household bargaining structures, social norms, and resource constraints may influence whether women's economic participation can be translated into improved nutritional outcomes (31–33). Therefore, examining married women's non-farm employment provides an important perspective for understanding how gendered socioeconomic transitions affect household dietary quality in rural China.

Existing research provides important insights into the determinants of household nutrition; however, several important gaps remain. First, previous studies have mainly focused on food consumption quantity, dietary diversity, or specific nutritional outcomes, while relatively limited attention has been paid to comprehensive dietary quality assessment that simultaneously considers insufficient intake, excessive intake, and overall dietary imbalance. This limitation is particularly important in the context of nutrition transition, where rural households may simultaneously experience nutritional inadequacy and emerging dietary imbalance (7). The China Dietary Balance Index-22 (DBI-22) provides a multidimensional framework for assessing these different aspects of dietary quality (34), but its application in examining socioeconomic determinants of rural household nutrition remains limited.

Second, although existing studies have examined the relationship between income growth, household resources, and dietary outcomes, less attention has been given to how women's employment transitions influence household dietary quality through gendered household mechanisms. Women's employment may affect nutrition not only by increasing household income but also by reshaping intra-household bargaining power, resource allocation patterns, and food-related decision-making processes. However, the specific role of married women's non-farm employment in shaping household nutritional welfare remains insufficiently understood, particularly in rural China.

Third, existing research has rarely examined whether the relationship between women's employment and dietary quality differs across different dimensions of nutrition outcomes and household contexts. Dietary improvements may not occur uniformly, as households differ in income constraints, nutritional demands, household life-cycle stages, and access to food markets. Therefore, further investigation is needed to identify the heterogeneous effects of women's employment on different dimensions of dietary quality and among different types of rural households.

To address these gaps, this study uses data from the China Rural Revitalization Survey (CRRS) to examine the relationship between married women's non-farm employment and household dietary quality in rural China. Unlike previous studies focusing mainly on food quantity or dietary diversity, this study adopts the DBI-22 framework to capture multiple dimensions of dietary quality, including excessive intake, insufficient intake, and overall dietary imbalance. Furthermore, this study investigates potential mechanisms linking women's employment transitions and dietary quality. Finally, this study examines heterogeneous effects across household conditions, including income levels, household life-cycle characteristics, and market constraints, offering evidence for designing gender-sensitive and nutrition-sensitive rural development policies.

## 2 Conceptual framework and research hypotheses

From the perspective of the social determinants of health framework, dietary outcomes are shaped by socioeconomic position, gender relations, household resources, and local living environments (3, 4). In rural households, women's labor participation represents a key channel through which socioeconomic transformation may affect household nutrition. According to the new household economics, households allocate labor, time, and income under resource constraints in order to maximize household utility (35). Women's transition from agricultural or unpaid household work to non-farm employment may therefore influence dietary outcomes through several interrelated mechanisms: income growth, intrahousehold bargaining and resource allocation, and changes in food consumption structure.

### 2.1 Women's employment transition and household dietary quality

Dietary quality is a multidimensional concept that involves not only whether households consume sufficient food but also whether their dietary patterns are balanced and consistent with recommended nutritional structures. According to the DBI-22 framework, dietary quality can be reflected through three dimensions: excessive intake measured by the High Bound Score (HBS), insufficient intake measured by the Low Bound Score (LBS), and overall dietary imbalance measured by the Diet Quality Distance (DQD) (34). Therefore, the influence of women's non-farm employment on household dietary quality may not be uniform across different dimensions.

From the perspective of household economics, women's transition from agricultural or unpaid household work to non-farm employment represents an important change in household resource allocation. According to Becker's (35) household production theory, households allocate labor, time, and income to maximize household welfare under resource constraints. Women's non-farm employment may increase household cash income, diversify livelihood sources, and improve access to food markets, thereby affecting household dietary outcomes. However, the specific effects may differ depending on whether employment mainly alleviates dietary constraints or promotes excessive food consumption.

First, women's non-farm employment is expected to have a stronger effect on reducing insufficient dietary intake (LBS). Rural households often face budget constraints and limited access to diverse foods, which may restrict the consumption of nutrient-rich foods such as meat, eggs, dairy products, fruits, and vegetables. Additional income generated through women's employment can relax household budget constraints and increase the ability to purchase diverse and nutritious foods. Therefore, women's employment may primarily improve dietary adequacy by reducing insufficient food intake.

Second, the effect of women's non-farm employment on excessive intake (HBS) is theoretically uncertain. On the one hand, increased income may expand food purchasing capacity and increase consumption of certain food categories. On the other hand, many rural households in China are still undergoing a transition from insufficient nutrition toward dietary improvement, and excessive consumption may not yet be the dominant nutritional challenge. Therefore, women's employment may not necessarily lead to significant increases in excessive dietary intake.

Third, women's non-farm employment may improve overall dietary balance (DQD) by promoting dietary diversification and upgrading household food consumption structures. Employment opportunities may expose women to broader information environments and consumption patterns while increasing their bargaining power in household decisions. These changes may encourage households to move away from monotonous staple-oriented diets toward more balanced dietary structures.

Based on the above theoretical analysis, we propose the following hypothesis:

**H1:** Married women's non-farm employment improves household dietary quality by reducing insufficient dietary intake and improving overall dietary balance, while its effect on excessive dietary intake remains uncertain.

### 2.2 Income growth pathway

Income is one of the most important determinants of household dietary outcomes. According to Bennett's (36) law, increases in household income generally lead to a transition from staple-oriented diets toward more diversified and higher-quality food consumption patterns. Existing studies have shown that income growth and income quality are closely associated with dietary diversity, nutritional demand, and food consumption upgrading among rural households (11, 14, 17, 37).

Compared with agricultural income, non-farm employment income is generally more stable and monetized, which can relax household budget constraints and improve households' ability to access diverse food products (22, 24). In rural households with limited economic resources, additional income generated from women's employment may first be used to increase the consumption of nutrient-rich foods, such as meat, eggs, dairy products, fruits, and vegetables. Therefore, income growth generated by women's non-farm employment is expected to primarily reduce insufficient dietary intake and improve the LBS dimension of dietary quality.

Meanwhile, income growth may also influence household food consumption structure and overall dietary balance. As households obtain greater economic capacity, they may gradually shift away from monotonous staple-based diets toward more diversified dietary patterns, thereby reducing deviations from recommended dietary structures and improving DQD. However, because rural households in China are still largely characterized by nutritional improvement rather than excessive consumption, income growth caused by women's employment may not necessarily lead to significant increases in excessive intake.

Therefore, income growth represents an important pathway through which women's non-farm employment affects multidimensional dietary quality.

**H2:** Married women's non-farm employment improves dietary adequacy and overall dietary balance through household income growth.

### 2.3 Household employment structure and resource allocation pathway

Beyond income effects, women's non-farm employment may influence household dietary quality by reshaping household employment structure and intra-household resource allocation. According to household bargaining theory, economic participation can enhance women's bargaining position and increase their involvement in household decision-making processes (27, 28). In rural households, where women often undertake responsibilities related to food preparation, childcare, and household nutrition management, changes in women's economic status may directly influence food-related decisions and resource allocation.

When women participate in non-farm employment, their contribution to household income and economic activities may increase their influence over household expenditure decisions. This may encourage greater allocation of household resources toward nutrition-related consumption, including higher-quality food products and more diversified dietary choices. Therefore, women's employment may improve dietary adequacy by reducing insufficient food intake (LBS) and enhance overall dietary balance (DQD).

However, the influence of women's employment on household dietary outcomes depends not only on whether women participate in employment, but also on how employment roles are distributed within the household. For example, households where both spouses participate in non-farm employment may have stronger economic capacity but may also face greater time constraints in food preparation. Therefore, the effect of women's employment on dietary quality may vary according to household employment structures and caregiving responsibilities.

Based on this perspective, this study further examines differences across household employment patterns, including households where neither spouse, only husband, only wife, or both spouses participate in non-farm employment.

**H3:** Married women's non-farm employment improves dietary adequacy and overall dietary balance by reshaping intra-household resource allocation.

### 2.4 Food consumption structure pathway

Women's non-farm employment may also influence household dietary quality by promoting changes in food consumption structure. During the nutrition transition process, rural households often gradually move from traditional grain-based diets toward more diversified diets containing higher proportions of animal products, fruits, vegetables, and other nutrient-rich foods (8, 9, 15).

Women's participation in non-farm employment may contribute to this transition through two channels. First, employment increases household economic resources and expands the range of affordable food choices. Second, women's exposure to broader social environments through employment may improve access to nutritional information and influence household food preferences.

Changes in food consumption structure are closely related to different dimensions of dietary quality. Increasing the proportion of nutrient-rich foods can reduce insufficient intake and improve dietary adequacy, thereby lowering LBS. Meanwhile, a more diversified food structure can reduce deviations from recommended dietary patterns and improve overall dietary balance, reflected by lower DQD. However, dietary structure upgrading does not necessarily imply excessive food consumption, especially in rural areas where nutritional improvement remains the primary challenge. Therefore, the influence of women's employment on HBS may remain limited.

Based on this pathway, we propose:

**H4:** Married women's non-farm employment improves household dietary quality by promoting food consumption structure upgrading, particularly through reducing dietary inadequacy and improving dietary balance.

### 2.5 Integrated conceptual framework

**Figure 1:**
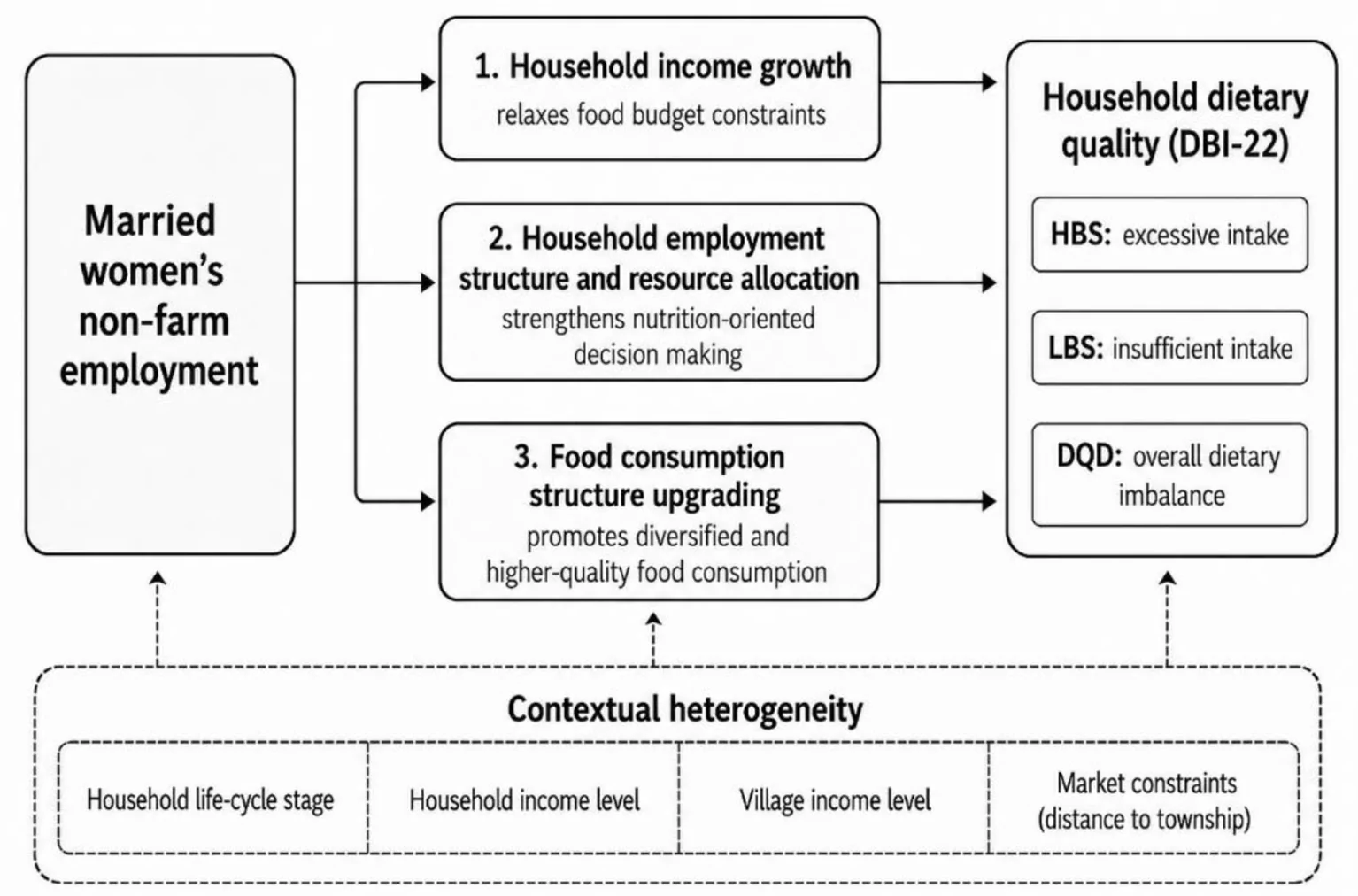
Conceptual framework of the association between married women's non-farm employment and household dietary quality.

Overall, this study conceptualizes married women's non-farm employment as a gendered socioeconomic transition that may influence household dietary quality through three main pathways. First, women's employment increases household income and relaxes budget constraints. Second, women's employment reshapes household employment structure and resource allocation. Third, women's employment promotes changes in food consumption structure. These pathways are not isolated; rather, they interact with household life-cycle stage, income level, and market constraints. Based on this framework, the empirical analysis examines not only the overall association between women's non-farm employment and dietary quality, but also pathway and subgroup differences. This design is consistent with empirical strategies that combine mechanism identification, robustness checks, and endogeneity correction in applied social science research (38, 39). The integrated conceptual framework is presented in Figure 1.

## 3 Materials and methods

### 3.1 Data source and study population

The data used in this study were obtained from the 2020 China Rural Revitalization Survey (CRRS), conducted by the Institute of Rural Development, Chinese Academy of Social Sciences. The CRRS is a nationally representative rural household survey designed to capture socioeconomic transformation and livelihood changes in rural China. The survey collects detailed information on household demographics, employment activities, income sources, food consumption behaviors, health-related conditions, and village-level characteristics.

Although more recent survey waves have become available, the 2020 CRRS was selected because it provides comprehensive household-level information required for this study, particularly detailed data on both women's employment status and household food consumption. These data allow us to construct multidimensional dietary quality indicators based on the China Dietary Balance Index-22 (DBI-22) and examine the mechanisms linking women's employment transitions with household nutritional outcomes. Compared with datasets that contain only employment or health information, the CRRS provides a unique combination of employment, consumption, and household characteristics, making it particularly suitable for investigating the relationship between women's non-farm employment and dietary quality.

The CRRS adopted a stratified sampling design covering different geographical regions and socioeconomic contexts. The survey covered 10 provinces, including Guangdong, Zhejiang, Shandong, Anhui, Henan, Sichuan, Guizhou, Shaanxi, Ningxia, and Heilongjiang, representing eastern, central, western, and northeastern China. The survey included 50 counties, 150 townships, and 304 administrative villages.

The original dataset was processed according to the following criteria. First, households without married women were excluded because this study focuses on the role of married women in household nutrition management and resource allocation. Second, observations with missing information on women's employment status, household food consumption, or key control variables were removed. After these procedures, the final analytical sample consisted of 1,723 rural households.

Since this study aims to identify the association between women's employment transition and household dietary quality rather than estimate long-term dynamic changes, a cross-sectional household survey with detailed information on employment and dietary consumption is appropriate for the research objective.

Although the analytical sample was obtained after restricting the survey to households containing married women and excluding observations with incomplete information, it retained broad geographical coverage across eastern, central, western, and northeastern China. Accordingly, the findings should be interpreted as evidence for the rural households represented in the analytical sample rather than as precise population estimates for all rural households in China.

### 3.2 Measures

#### 3.2.1 Outcome variable: household dietary quality

The primary outcome variable was household dietary quality, which was evaluated using the China Dietary Balance Index-2022 (DBI-22) (34). Compared with traditional indicators focusing only on food consumption quantity or dietary diversity, the DBI-22 provides a multidimensional assessment of dietary balance by simultaneously considering insufficient intake, excessive intake, and overall dietary imbalance. This multidimensional measurement is suitable for capturing the coexistence of inadequate intake, dietary imbalance, and excessive intake during nutrition transitions (5–7).

According to the DBI-22 scoring system, this study constructed three indicators:

First, the High Bound Score (HBS) reflects excessive dietary intake. A higher HBS indicates a greater degree of excessive food consumption.

Second, the Low Bound Score (LBS) captures insufficient dietary intake. A higher absolute value of LBS indicates a more serious degree of insufficient dietary intake.

Third, the Diet Quality Distance (DQD) measures the overall imbalance of dietary structure. A higher DQD indicates a greater deviation from recommended dietary patterns and poorer dietary quality.

The DBI-22 scoring criteria are shown in Table 1.

**Table 1:** Scoring criteria of the China dietary balance index (DBI-22).

DBI Score	Good	Acceptable	Mild	Moderate	Severe
HBS	0	1–9	10–18	19–27	>27
LBS	0	1–14	15–29	29–43	>43
DQD	0	1–19	20–38	39–57	>57

#### 3.2.2 Exposure variable: married women's non-farm employment

The primary exposure variable was married women's participation in non-farm employment.

Based on employment information from the CRRS questionnaire, women were classified as participating in non-farm employment if they engaged in either:

(1) exclusive non-farm employment activities; or
(2) concurrent agricultural and non-farm employment activities.

Women who were unemployed or engaged only in agricultural activities were classified as not participating in non-farm employment.

This definition reflects the characteristics of rural livelihood diversification in China, where many households combine agricultural production with off-farm employment as part of their income strategies. Therefore, including both exclusive non-farm employment and concurrent non-farm activities allows the measure to capture broader employment transitions from traditional agricultural activities toward diversified livelihood patterns.

The variable was coded as 1 for participation in non-farm employment and 0 otherwise.

#### 3.2.3 Pathway variables

Based on the conceptual framework and prior studies on income, gendered resource allocation, and food consumption behavior, this study examined three potential pathways linking married women's non-farm employment and household dietary quality (27, 28, 35, 36).

First, household income was used to represent the economic resource pathway. Increased income may improve households' ability to purchase diverse and nutritious foods, as shown in studies on income growth, food consumption, and dietary diversity in rural households (11, 14, 15, 25).

Second, household employment structure was used to capture the intra-household resource allocation pathway. Women's participation in non-farm employment may change the relative economic contributions and decision-making roles of household members, thereby affecting food-related resource allocation and nutrition-oriented household spending (27, 28, 30, 40).

Third, food consumption structure was considered as a dietary transition pathway. This pathway reflects whether women's employment promotes changes in household food choices toward more nutritious food categories and higher-quality dietary patterns, which is consistent with evidence on food consumption upgrading and rural food environments (9, 12, 13). These pathway variables were selected because they may influence different dimensions of dietary quality, particularly insufficient intake and overall dietary imbalance.

#### 3.2.4 Covariates

To reduce potential confounding effects, this study controlled for individual-, household-, and village-level characteristics that may influence household dietary quality. These covariates were selected based on previous evidence that dietary outcomes are associated with demographic structure, income, education, household composition, food markets, and local access conditions (8, 13, 14, 17, 41).

Individual-level covariates included: age of household head, education level of household head, women's age, women's education level, whether the household head held a village cadre position.

Household-level covariates included: cultivated land area, household size, number of laborers.

Village-level covariates included: village per capita disposable income, distance between village committee and township government, registered village population. Descriptive statistics of the DBI-22 outcome variables are presented in Table 2, while definitions and descriptive statistics of the study variables are presented in Table 3.

**Table 2:** Descriptive statistics of the DBI-22 outcome variables.

Variable	Mean	Std. Dev.	Min	Max	Skewness	Kurtosis
HBS	12.795	4.554	0.000	20.000	−0.934	3.758
LBS	10.513	5.716	0.000	32.000	0.124	2.600
DQD	23.308	7.080	2.630	42.000	−0.241	2.565

**Table 3:** Definitions and descriptive statistics of variables.

Variable	Definition	Mean	Std. Dev.
Married women's non-farm employment	Equals 1 if the married woman is in non-farm employment or concurrent agricultural and non-farm employment; 0 otherwise	0.295	0.456
Age of household head	Age of household head	52.617	8.255
Education of household head	No schooling = 0, primary school = 6, junior high school = 9, senior high school/technical secondary school/vocational high school/technical school = 12, junior college = 15, bachelor's degree = 16, postgraduate = 19	8.193	2.861
Woman's age	Wife's age	51.352	8.027
Woman's education	No schooling = 0, primary school = 6, junior high school = 9, senior high school/technical secondary school/vocational high school/technical school = 12, junior college = 15, bachelor's degree = 16, postgraduate = 19	7.239	3.240
Whether the household head holds a village position	Yes = 1, No = 0	0.189	0.391
Total household income (log)	Total household income in 2019 (yuan), logged	10.733	0.959
Total cultivated land area	Actual cultivated land area of the household (mu)	20.339	58.897
Household size	Number of household members	4.278	1.349
Number of household laborers	Number of laborers in the household	3.237	0.918
Village per capita disposable income in 2019 (log)	Village per capita disposable income in 2019 (yuan), logged	9.404	0.655
Distance from village committee to township government (km)	Distance from village committee to township government (km)	5.580	5.836
Total registered households in the village	Total registered households in the village	692.227	460.816
Food consumption structure	Share of meat, egg, and dairy expenditure in total food expenditure	0.590	0.267

The three DBI-22 indicators did not exhibit severe distributional abnormalities. The skewness values ranged from −0.934 to 0.124, while kurtosis ranged from 2.565 to 3.758, suggesting no extreme skewness or concentration. Because the baseline models estimate conditional mean associations, strict normality of the outcome variables is not required; robust standard errors were used to improve inference in the presence of potential heteroskedasticity.

### 3.3 Statistical analysis

#### 3.3.1 Baseline model

To examine the association between married women's non-farm employment and household dietary quality, the baseline regression model was specified as Equation 1:


$$\begin{array}{c} DBI_{i}=\alpha_{0}+\alpha_{1}NFE_{i}+\underset{j}{\sum }\alpha_{j}Control_{ij}+\lambda_{p}+\varepsilon_{i} \end{array}$$ (1)

where *DBI*_*i*_ represents household dietary quality indicators, including HBS, LBS, and DQD.

*NFE*_*i*_ represents whether married women participated in non-farm employment.

*Control*_*i*_ denotes a set of individual-, household-, and village-level covariates.

λ_*p*_ represents province fixed effects.

ε_*i*_ is the random error term.

The coefficient α_1_ captures the association between married women's non-farm employment and household dietary quality.

#### 3.3.2 Pathway analysis

To explore potential pathways underlying the observed association between married women's non-farm employment and household dietary quality, exploratory pathway analyses were conducted. Based on the conceptual framework, three dimensions were examined: household income, household employment structure and resource allocation, and food consumption structure. These analyses were designed to assess whether the observed empirical patterns were consistent with the proposed theoretical pathways rather than to establish formal causal mediation effects.

Based on the conceptual framework, this study examined whether women's non-farm employment affected three pathway variables: household income; household employment structure and resource allocation; food consumption structure.

The pathway model was specified as Equation 2:


$$\begin{array}{c} M_{i}=\beta_{0}+\beta_{1}NFE_{i}+\underset{j}{\sum }\beta_{j}Control_{ij}+\lambda_{p}+\mu_{i} \end{array}$$ (2)

where *M*_*i*_ represents the pathway variables, including household income, household employment structure, and food consumption structure.

*NFE*_*i*_ represents married women's participation in non-farm employment.

*Control*_*i*_, λ_*p*_, and μ_*i*_ have the same meanings as described above.

The coefficient β_1_ reflects whether women's employment transition significantly influences each potential pathway.

#### 3.3.3 Endogeneity and sensitivity analyses

Because women's participation in non-farm employment is a self-selected decision rather than a randomly assigned treatment, potential endogeneity concerns may arise from omitted variables and unobservable household characteristics.

To address these concerns, this study first applies instrumental variable estimation using the non-farm employment rate of other women within the same village (excluding the sample household) as an instrument. This instrument captures local labor market networks and peer employment environments that influence women's employment decisions.

Furthermore, considering that the endogenous treatment variable is binary, we additionally employ the Conditional Mixed Process (CMP) estimator as a robustness check. The CMP approach jointly estimates the endogenous treatment equation and the outcome equation while allowing correlation between error terms, making it suitable for models involving binary endogenous variables.

Propensity score matching was additionally employed to reduce observable differences between households with and without married women participating in non-farm employment. Propensity scores were estimated using the covariates included in the baseline model. One-to-one nearest-neighbor matching, one-to-four matching, radius matching, and kernel matching were conducted. Covariate balance was evaluated using standardized bias, pseudo-R^2^, and the likelihood-ratio statistic.

As additional sensitivity checks, the DBI-22 outcomes were alternatively transformed into ordered dietary-quality categories and estimated using ordered Probit models. Key continuous variables were also winsorized to examine whether the baseline estimates were sensitive to extreme observations.

#### 3.3.4 Heterogeneity analysis

To examine whether the relationship between women's non-farm employment and household dietary quality varies across different household contexts, subgroup analyses were conducted according to household income levels, life-cycle characteristics, and market constraints.

In addition to comparing coefficients across groups, Wald tests were performed to formally examine whether estimated coefficients differ statistically between subgroups.

## 4 Results

### 4.1 Baseline association between married women's non-farm employment and household dietary quality

The baseline regression results examining the association between married women's non-farm employment and household dietary quality are presented in Table 4. Household dietary quality is measured using three DBI-22 indicators, including the High Bound Score (HBS), Low Bound Score (LBS), and Diet Quality Distance (DQD), which capture excessive dietary intake, insufficient dietary intake, and overall dietary imbalance, respectively (34).

**Table 4:** Association between married women's non-farm employment and household dietary quality.

Variable	(1)/(HBS)	(2)/(HBS)	(3)/(LBS)	(4)/(LBS)	(5)/(DQD)	(6)/(DQD)
Married women's non-farm employment	−0.045/(0.233)	−0.134/(0.249)	−2.641^***^/(0.295)	−1.616^***^/(0.314)	−2.687^***^/(0.355)	−1.749^***^/(0.379)
Age of household head		−0.010/(0.014)		0.072^***^/(0.018)		0.061^***^/(0.022)
Education of household head		0.042/(0.041)		−0.126^**^/(0.052)		−0.084/(0.063)
Woman's age		0.036^*^/(0.019)		−0.054^**^/(0.024)		−0.019/(0.029)
Woman's education		0.037/(0.039)		−0.170^***^/(0.049)		−0.132^**^/(0.059)
Whether the household head holds a village position		0.433/(0.277)		−0.726^**^/(0.350)		−0.294/(0.421)
Total cultivated land area		−0.010^***^/(0.002)		0.003/(0.002)		−0.007^**^/(0.003)
Household size		−0.426^***^/(0.095)		−0.278^**^/(0.120)		0.148/(0.144)
Number of household laborers		−0.261^*^/(0.142)		0.239/(0.180)		−0.021/(0.217)
Village per capita disposable income in 2019 (log)		0.214/(0.173)		−1.066^***^/(0.219)		−0.852^***^/(0.264)
Distance to township government		−0.015/(0.019)		0.076^***^/(0.024)		0.061^***^/(0.028)
Total registered households in the village		– < 0.001/(< 0.001)		−0.001^**^/(< 0.001)		−0.001^***^/(< 0.001)
Province	Controlled	Controlled	Controlled	Controlled	Controlled	Controlled
Constant	12.900^***^ (0.132)	1.791 (2.120)	11.240^***^ (0.166)	29.120^***^/(2.678)	24.140^***^ (0.200)	30.910^***^ (3.227)
Observations	1,723	1,723	1,723	1,723	1,723	1,723
*R^2^*	< 0.001	0.098	0.045	0.138	0.032	0.125

^***^, ^**^, and ^*^ indicate significance at the 1%, 5%, and 10% levels, respectively; robust standard errors are reported in parentheses.

The results show that married women's non-farm employment is significantly associated with improved household dietary quality. Specifically, women's non-farm employment is negatively associated with LBS and DQD, indicating that employment participation reduces insufficient dietary intake and improves overall dietary balance.

The significant reduction in LBS suggests that women's employment helps households overcome constraints related to food accessibility and affordability. Additional employment income may increase households' ability to purchase diverse and nutrient-rich foods, thereby improving dietary adequacy. Meanwhile, the negative association with DQD indicates that women's employment contributes to optimizing household dietary structures and reducing deviations from recommended dietary patterns.

However, the association between women's non-farm employment and HBS was not statistically significant. This finding does not necessarily indicate that women's employment has no relationship with excessive dietary intake. Instead, it suggests that the overall effect of employment participation on excessive intake may be limited because different pathways may operate simultaneously and offset each other. For rural households undergoing nutrition transition, employment-related income growth may increase food purchasing capacity, while improved nutritional awareness and consumption structure upgrading may simultaneously encourage healthier dietary choices. Therefore, the net effect on excessive intake may remain statistically insignificant. This result is consistent with the current stage of rural nutrition transition in China, where improving dietary adequacy and balance remains a more prominent challenge than widespread overconsumption (5–7).

### 4.2 Sensitivity analyses

To examine the stability of the baseline findings, several sensitivity analyses were conducted.

First, considering the ordered characteristics of dietary quality classification, ordered Probit models were used as an alternative estimation approach. The results are presented in Table 5.

**Table 5:** Sensitivity analysis using ordered Probit models.

Variable	HBS	LBS	DQD
Married women's non-farm employment	0.114/ (0.070)	0.366^***^/ (0.071)	0.221^***^/ (0.077)
Other control variables	Controlled	Controlled	Controlled
Region	Controlled	Controlled	Controlled
Observations	1,723	1,723	1,723

In the ordered Probit analysis, higher ordered categories indicate better dietary quality after reverse coding.

^***^Indicates significance at the 1% level; robust standard errors are reported in parentheses.

The results remained consistent with the baseline findings, suggesting that the association between married women's non-farm employment and household dietary quality was not sensitive to model specification.

Second, to reduce the potential influence of extreme values, key continuous variables were winsorized, and the model was re-estimated. The results are shown in Table 6.

**Table 6:** Sensitivity analysis after adjusting extreme observations.

Variable	HBS	LBS	DQD
Married women's non-farm employment	−0.314/ (0.249)	−1.616^***^/ (0.314)	−1.552^***^/ (0.355)
Other control variables	Controlled	Controlled	Controlled
Region	Controlled	Controlled	Controlled
Constant	1.791/ (2.120)	29.120^***^/ (2.678)	30.990^***^/ (3.022)
Observations	1,723	1,723	1,723
*R^2^*	0.098	0.138	0.126

^***^Indicates significance at the 1% level; robust standard errors are reported in parentheses.

After adjusting for extreme values, the main conclusions remained unchanged, further supporting the robustness of the relationship between women's non-farm employment and household dietary quality.

### 4.3 Addressing potential endogeneity

Although multiple covariates were controlled, women's participation in non-farm employment may still be affected by self-selection and unobserved factors. Therefore, instrumental variable estimation and propensity score matching were applied to address potential endogeneity concerns.

#### 4.3.1 Instrumental variable estimation

The non-farm employment rate of other women within the same village was used as an instrumental variable for married women's non-farm employment. This instrument reflects the role of local labor-market networks and peer employment environments in shaping women's employment decisions, while reducing direct household-level confounding (39). The estimation results are presented in Table 7.

**Table 7:** Instrumental variable estimation results.

Variable	First stage/married women's non-farm employment	Second stage/dietary quality HBS	Second stage/dietary quality LBS	Second stage/dietary quality DQD
Proportion of non-farm employment among other villagers excluding the sample household	0.210^***^/(0.054)			
Whether the woman is in non-farm employment		−7.521/(5.889)	−7.530^**^/(3.581)	−13.786^***^/(4.781)
Other control variables	Controlled	Controlled	Controlled	Controlled
Region	Controlled	Controlled	Controlled	Controlled
Observations	1,723	1,723	1,723	1,723
Kleibergen-Paap rk LM	14.820^***^			
First-stage F statistic	15.060^***^			

The Kleibergen–Paap rk LM statistic tests the null hypothesis that the model is underidentified. Rejection of the null indicates that the instrumental-variable equation satisfies the identification requirement. The first-stage F statistic is reported to assess instrument relevance and strength.

^***^ and ^**^ indicate significance at the 1% and 5% levels, respectively; robust standard errors are reported in parentheses.

The Kleibergen–Paap rk LM statistic was 14.820 (*p* < 0.01), rejecting the null hypothesis of underidentification. The first-stage F statistic was 15.060, further supporting the relevance of the instrument.

The instrumental variable results were consistent with the baseline findings, indicating that after accounting for potential endogenous selection, married women's non-farm employment remained significantly associated with better household dietary quality.

#### 4.3.2 Propensity score matching

Propensity score matching (PSM) was further applied to reduce observable differences between households with and without women participating in non-farm employment.

The balance test results are shown in Table 8.

**Table 8:** Covariate balance test after propensity score matching.

Matching method	Pseudo R^2^	LR statistic	Standardized bias (%)
Before matching	0.191	412.02	25.9
One-to-one	0.010	14.350	5.7
One-to-four	0.004	5.810	4.0
Radius matching	0.003	4.180	3.2
Kernel matching	0.004	5.880	4.0

After matching, differences in observable characteristics between the treatment and control groups were substantially reduced, indicating that the matching procedure achieved good balance.

The average treatment effects estimated using different matching methods are reported in Table 9.

**Table 9:** Average treatment effects of women's non-farm employment after matching.

Matching method	HBS	HBS	HBS	LBS	LBS	LBS	DQD	DQD	DQD
Matching method	Average treatment effect	Standard error	*t*-value	Average treatment effect	Standard error	*t*-value	Average treatment effect	Standard error	*t*-value
One-to-one	−0.536^**^	0.272	−1.969	−1.418^***^	0.438	−3.240	−1.663^***^	0.500	−3.330
One-to-four	−0.348	0.231	−1.507	−1.476^***^	0.384	−3.840	−1.569^***^	0.433	−3.620
Radius matching	−0.409^*^	0.212	−1.925	−1.575^***^	0.351	−4.490	−1.605^***^	0.397	−4.040
Kernel matching	−0.411^*^	0.212	−1.940	−1.528^***^	0.370	−4.130	−1.603^***^	0.418	−3.830
Average	−0.426			−1.499			−1.610		

^***^, ^**^, and ^*^ indicate significance at the 1%, 5%, and 10% levels, respectively; robust standard errors are reported in parentheses.

The PSM results further confirmed that married women's non-farm employment was positively associated with household dietary quality improvement, supporting the reliability of the baseline results.

#### 4.3.3 Conditional mixed process estimation

To further examine whether the baseline findings remain robust after accounting for endogenous selection into non-farm employment, the CMP estimator was applied. The results are reported in Table 10.

**Table 10:** Conditional mixed process (CMP) estimation results.

Variables	First stage	(1) HBS	(2) LBS	(3) DQD
Village-level female non-farm employment rate	0.864^***^ (0.142)			
Married women's non-farm employment		−0.176 (0.265)	−1.936^***^ (0.437)	−2.145^***^ (0.548)
atanhrho		0.083 (0.091)	−0.327^**^ (0.104)	−0.386^***^ (0.118)
Controls		Yes	Yes	Yes
Province FE		Yes	Yes	Yes
N		1,723	1,723	1,723

^***^ and ^**^ indicate significance at the 1% and 5% levels, respectively; robust standard errors are reported in parentheses.

The CMP results are reported in Table 10. The estimated coefficients remain consistent with the baseline regression and instrumental variable results. Specifically, married women's non-farm employment continues to show a significant negative association with LBS and DQD, indicating that women's employment participation is associated with reduced insufficient dietary intake and improved overall dietary balance. Meanwhile, the coefficient for HBS remains statistically insignificant, suggesting that women's employment does not generate a substantial overall increase in excessive dietary intake.

The estimated error correlations are statistically significant for the LBS and DQD equations, indicating endogenous selection in these two dietary outcomes, whereas no statistically significant error correlation is observed for HBS. This finding further confirms the necessity of accounting for self-selection into non-farm employment and supports the reliability of the CMP estimates.

Overall, the CMP results provide additional evidence that the relationship between married women's non-farm employment and household dietary quality is robust after considering potential endogeneity caused by employment selection.

### 4.4 Subgroup analyses

To explore whether the relationship between women's non-farm employment and dietary quality differed across household and community contexts, subgroup analyses were conducted according to household life-cycle stages, economic conditions, and village characteristics. These subgroup dimensions were selected because household life-cycle stage, intergenerational care responsibilities, income conditions, and market access can shape consumption priorities and dietary outcomes (13, 42–44).

The classification criteria of household life-cycle stages are presented in Table 11.

**Table 11:** Classification of household life-cycle stages.

Household life cycle	Stage characteristics	Observations
Burden stage	Both the old-age dependency ratio and child dependency ratio are non-zero	297
Child-rearing stage	Old-age dependency ratio = 0, child dependency ratio ≠ 0	621
Older-adult-support stage	Old-age dependency ratio ≠ 0, child dependency ratio = 0	247
Stable stage	Both the old-age dependency ratio and child dependency ratio are 0	558

Based on these classifications, subgroup regression analyses were conducted, and the results are reported in Table 12.

**Table 12:** Subgroup analysis of the association between women's non-farm employment and household dietary quality.

Heterogeneity dimension	Group	HBS	LBS	DQD	Observations
Income heterogeneity (village)	Low village income	−0.673^**^ (0.319)	−1.613^***^ (0.545)	−2.286^***^ (0.733)	867
	High village income	−0.135 (0.188)	−1.080^*^ (0.635)	−1.215^*^ (0.693)	856
Income heterogeneity (household)	Low income	−1.600^**^ (0.705)	−0.866 (0.669)	−2.154^***^ (0.735)	430
	Lower-middle income	0.239 (0.310)	−2.284^***^ (0.640)	−1.441^*^ (0.760)	431
	Upper-middle income	−0.245 (0.408)	−1.979^***^ (0.641)	−1.949^***^ (0.693)	431
	High income	0.170 (0.382)	−1.028^*^ (0.615)	−0.603 (0.702)	431
Life-cycle heterogeneity	Burden stage	0.499 (0.681)	−1.989^**^ (0.820)	−1.270 (0.825)	297
	Child-rearing stage	−0.544^*^ (0.317)	−1.409^***^ (0.517)	−1.437^**^ (0.593)	621
	Older-adult-support stage	−0.617/(0.716)	−2.826^***^ (0.826)	−2.778^***^ (0.949)	247
	Stable stage	−0.642/(0.493)	−1.052^*^ (0.560)	−1.198^*^ (0.661)	558
Market-constraint heterogeneity	Closer to township	−0.203 (0.170)	−1.292^**^ (0.542)	−1.495^***^ (0.552)	905
	Farther from township	−0.754^**^ (0.355)	−1.290^**^ (0.607)	−2.044^**^ (0.818)	818

^***^, ^**^, and ^*^ indicate significance at the 1%, 5%, and 10% levels, respectively; robust standard errors are reported in parentheses.

The results indicate that the association between women's non-farm employment and dietary quality varied across different household groups. The improvement effect was more pronounced among households with stronger nutritional demands, relatively disadvantaged economic conditions, and limited external resources. These patterns are consistent with studies showing that household composition, childcare and older-adult care responsibilities, and local food environments influence dietary behavior and nutrition outcomes (13, 16, 41, 45). Related evidence also indicates that household nutrition and welfare are shaped by child health interventions, gendered labor division, women's empowerment, healthy-diet policies, access to finance, caregiving responsibilities, and women's access to productive resources (46–52).

These findings suggest that women's employment participation may have greater nutritional benefits among households facing higher vulnerability, highlighting its potential role in reducing nutrition-related inequalities. This interpretation is consistent with the social determinants of health perspective, which emphasizes that socioeconomic resources and household conditions jointly shape health-related behaviors (3, 4).

### 4.5 Pathway analyses

It should be noted that the pathway analysis does not necessarily imply that each mechanism generates effects in the same direction as the baseline association. The total effect estimated in the baseline model reflects the combined outcome of multiple mechanisms. Therefore, some specific pathways may significantly influence HBS even when the overall association between women's non-farm employment and HBS is statistically insignificant.

Based on the conceptual framework, pathway analyses were conducted to further explore how married women's non-farm employment was associated with improvements in household dietary quality. Three potential pathways were examined: household income, household resource allocation, and food consumption structure. These pathways were grounded in household economics, nutrition transition theory, and gendered resource allocation perspectives (27, 35, 36, 38).

#### 4.5.1 Household income pathway

The results regarding the household income pathway are shown in Table 13.

**Table 13:** Household income pathway analysis.

Variable	Household income	HBS	LBS	DQD
Married women's non-farm employment	0.239^**^/(0.053)	−0.454^*^/(0.252)	−1.775^***^/(0.312)	−2.229^***^ (0.387)
Household income		0.305^***^/(0.115)	−0.737^***^ (0.143)	−0.432^**^/(0.175)
Other control variables	Controlled	Controlled	Controlled	Controlled
Observations	1,723	1,723	1,723	1,723
*R^2^*	0.076	0.054	0.132	0.084

^***^, ^**^, and ^*^ indicate significance at the 1%, 5%, and 10% levels, respectively; robust standard errors are reported in parentheses.

The results indicate that married women's participation in non-farm employment significantly increased household income levels. Increased economic resources may improve households' ability to purchase diverse and nutritious foods, thereby contributing to better dietary quality. This is consistent with Bennett's law and empirical evidence that income growth and non-farm income improve food security and dietary quality (11, 14, 25, 36).

#### 4.5.2 Household resource allocation pathway

The results for household resource allocation are presented in Table 14.

**Table 14:** Household resource allocation pathway analysis.

Variable	HBS	LBS	DQD
Husband only employed	0.142 (0.186)	0.209 (0.279)	0.351 (0.359)
Wife only employed	−0.079 (0.387)	−1.358^*^ (0.706)	−1.437^**^ (0.726)
Both spouses employed	−0.474^*^ (0.250)	−1.098^**^ (0.493)	−1.571^**^ (0.666)
Husband's age	−0.003 (0.010)	0.067^***^ (0.019)	0.065^***^ (0.021)
Husband's years of schooling	0.004 (0.029)	−0.121^**^ (0.051)	−0.116^*^ (0.067)
Wife's age	0.027^**^ (0.012)	−0.042 (0.033)	−0.015 (0.039)
Wife's years of schooling	0.054^**^ (0.027)	−0.204^***^ (0.068)	−0.151^**^ (0.072)
Whether holding a village position	0.156 (0.380)	−0.794^*^ (0.413)	−0.638 (0.591)
Cultivated land area (log)	0.077 (0.105)	−0.534^***^ (0.206)	−0.457^*^ (0.234)
Household size	0.277^***^ (0.090)	−0.345^**^ (0.136)	−0.068 (0.195)
Number of laborers	−0.210 (0.128)	0.203 (0.137)	−0.006 (0.208)
Village per capita disposable income (log)	−0.140 (0.185)	−0.317 (0.353)	−0.457 (0.340)
Distance to township government (km)	0.036^*^ (0.021)	0.020 (0.021)	0.056^*^ (0.032)
Total registered households	– < 0.001 (< 0.001)	−0.001^**^ (< 0.001)	−0.001^**^ (< 0.001)
Province fixed effects	Yes	Yes	Yes
Observations	1,723	1,723	1,723
*R^2^*	0.196	0.201	0.171
Adjusted *R^2^*	0.185	0.190	0.160

^***^, ^**^, and ^*^ indicate significance at the 1%, 5%, and 10% levels, respectively; robust standard errors are reported in parentheses.

The findings suggest that women's non-farm employment changed intra-household employment patterns and enhanced women's contribution to household resources, which may increase their involvement in food-related decision-making and promote healthier dietary choices. This aligns with evidence that women's empowerment and control over resources can improve household food security, dietary diversity, and nutritional welfare, although such effects depend on household bargaining conditions (27, 28, 30, 31, 40).

#### 4.5.3 Food consumption structure pathway

The relationship between women's non-farm employment and household food consumption structure is presented in Table 15.

**Table 15:** Food consumption structure pathway analysis.

Variable	Food consumption structure	HBS	LBS	DQD
Married women's non-farm employment	0.040^***^/(0.014)	−0.569^**^/(0.249)	−1.505^***^ (0.298)	−2.074^***^ (0.380)
Food consumption structure		2.885^***^/(0.420)	−6.779^***^ (0.502)	−3.893^***^ (0.641)
Other control variables	Controlled	Controlled	Controlled	Controlled
Observations	1,723	1,723	1,723	1,723
*R^2^*	0.114	0.080	0.216	0.104
Specification note	Mediator equation	Outcome equation	Outcome equation	Outcome equation

^***^ and ^**^ indicate significance at the 1% and 5% levels, respectively; robust standard errors are reported in parentheses.

The results show that women's participation in non-farm employment promoted changes in household food consumption structure, especially by increasing the consumption of more diverse and nutritious food categories. This is consistent with studies on food consumption upgrading, income-driven dietary change, and the role of food environments in shaping nutrition outcomes (9, 12, 13, 15).

Overall, these pathway analyses suggest that women's employment transitions may improve specific dimensions of household dietary quality through economic resources, intra-household allocation, and dietary behavior pathways. These findings connect women's employment to the broader public health literature on socioeconomic resources, gendered household dynamics, and nutrition-related inequality (2–4).

## 5 Discussion

### 5.1 Principal findings

This study examined the relationship between married women's non-farm employment and household dietary quality using nationally representative data from the China Rural Revitalization Survey (CRRS). Household dietary quality was comprehensively measured using the DBI-22 index, capturing both insufficient intake, excessive intake, and overall dietary imbalance.

The main findings are threefold. First, married women's non-farm employment is significantly associated with improved household dietary quality, mainly reflected in reductions in insufficient dietary intake and overall dietary imbalance, while no significant effect is observed for excessive intake. Second, the results remain robust after a series of sensitivity analyses, including alternative model specifications and propensity score matching. Third, pathway analyses suggest that this relationship operates through income growth, intra-household resource allocation, and food consumption structure upgrading.

These findings suggest that women's employment transitions are not only an economic phenomenon, but also an important driver of household nutritional welfare.

### 5.2 Interpretation from a public health perspective

From a public health perspective, these results highlight the importance of women's employment as a social determinant of health. Dietary quality is increasingly recognized as a key component of population health, closely linked to chronic disease risks, nutritional inequality, and long-term human capital development.

The finding that women's non-farm employment primarily reduces insufficient dietary intake rather than excessive intake reflects the current stage of nutritional transition in rural China. Rural households are still in a phase where under-consumption of diverse and high-quality foods remains a more prominent issue than over-consumption. Therefore, additional household income and improved decision-making capacity are more likely to be directed toward improving dietary adequacy rather than increasing dietary excess.

This also implies that improvements in rural dietary quality are closely tied to socioeconomic empowerment processes rather than only food availability.

### 5.3 Comparison with existing literature

The findings of this study are consistent with existing literature emphasizing the importance of women's empowerment and income in improving household nutrition. Previous studies have shown that women's decision-making power and economic participation are positively associated with food security and dietary diversity.

However, this study extends the literature in three important ways. First, it shifts the focus from food consumption quantity or dietary diversity to comprehensive dietary quality using the DBI-22 index. Second, it provides evidence from a large-scale nationally representative rural dataset in China, contributing micro-level evidence to the global literature on gender and nutrition. Third, it integrates multiple pathways, showing that the effect of women's employment is not solely driven by income but also by intra-household allocation and behavioral changes in food consumption.

Unlike previous studies that mainly focus on food quantity or dietary diversity, this study demonstrates that women's employment affects different dimensions of dietary quality differently. The absence of a significant effect on HBS suggests that economic improvement does not automatically translate into excessive consumption, while significant reductions in LBS and DQD indicate that employment transitions mainly facilitate dietary improvement among rural households still experiencing nutritional constraints.

### 5.4 Policy implications

These findings have several implications for public health and rural development policies.

First, promoting women's participation in non-farm employment may serve as an effective strategy for improving household dietary quality and reducing nutrition-related inequalities in rural areas. Employment policies targeting rural women, such as vocational training and labor market access support, may generate broader health benefits beyond income growth.

Second, improving dietary quality requires attention not only to income levels but also to intra-household decision-making structures. Strengthening women's bargaining power within households may enhance the nutritional efficiency of household resource allocation.

Third, public health interventions in rural areas should integrate economic and nutritional perspectives, recognizing that dietary behaviors are shaped by both income constraints and gendered household dynamics.

### 5.5 Limitations and future research

This study has several limitations. First, although instrumental variable and propensity score matching methods were used, the cross-sectional nature of the data limits causal interpretation. Future research using panel data or quasi-experimental designs would help strengthen causal inference.

Second, dietary quality is measured at the household level, which may mask intra-household heterogeneity in dietary intake among different members such as children, women, and older adults.

Third, while this study examines income, resource allocation, and food consumption structure as key pathways, other mechanisms such as nutritional knowledge, time use, and food environment accessibility should be further explored.

## 6 Conclusion

This study examined the association between married women's non-farm employment and household dietary quality using nationally representative data from the China Rural Revitalization Survey (CRRS). Household dietary quality was comprehensively measured using the China Dietary Balance Index (DBI-22), capturing insufficient intake, excessive intake, and overall dietary imbalance.

The empirical results indicate that married women's participation in non-farm employment is significantly associated with improved household dietary quality. Specifically, women's employment reduces insufficient dietary intake and overall dietary imbalance, while showing no significant effect on excessive intake. These findings are robust across multiple model specifications, instrumental variable estimation, and propensity score matching analyses.

Further analysis suggests that the relationship operates through multiple pathways, including increased household income, improved intra-household resource allocation, and changes in food consumption structure. These mechanisms highlight the important role of women's employment transitions in shaping household nutritional outcomes.

From a public health perspective, the findings suggest that promoting women's participation in non-farm employment may contribute to improving dietary quality and reducing nutrition-related inequalities in rural populations. Policies aimed at enhancing gender-inclusive labor market participation may therefore have important implications for population health and nutritional welfare.

This study has limitations. The use of cross-sectional data restricts causal interpretation, and future research based on longitudinal data is needed to further validate the dynamic effects of women's employment on dietary outcomes.

## Data Availability

The data analyzed in this study is subject to the following licenses/restrictions. The CRRS dataset is not publicly available due to data use agreements and confidentiality restrictions. Access requires permission from the data provider, the Institute of Rural Development, Chinese Academy of Social Sciences. Requests to access these datasets should be directed to http://rdi.cssn.cn/dcsj/.
